# A novel nanoparticle glutathione and *Lepidium sativum* treatment for gentamicin-induced acute renal failure in rats

**DOI:** 10.1038/s41598-025-05385-3

**Published:** 2025-06-20

**Authors:** Mahmoud S. Sabra, Essmat A. H. Allam, Madeha H. A. Darwish, Enas A. Abdelhafez, Abeer S. Hassan, Ahmed A. N. Ahmed, Hoda S. Sherkawy, Marwa G. Gamea

**Affiliations:** 1 Department of Pharmacology, Faculty of Veterinary Medicine, Assiut University, Assiut, 71526 Egypt; 2 Department of Pharmacology and Toxicology, Faculty of Pharmacy, Assiut University, Assiut, 71526 Egypt; 3 Department of Animal and Poultry Behavior and Management, Faculty of Veterinary Medicine, Assiut University, Assiut, 71516 Egypt; 4 Department of Cell and Tissues, Faculty of Veterinary Medicine, Assiut University, Assiut, 71526 Egypt; 5 Department of Pharmaceutics, Faculty of Pharmacy, South Valley University, Qena, 83523 Egypt; 6 Pharmacology Department, Faculty of Medicine, Al-Azhar University, Assiut Branch, Assiut, 71526 Egypt; 7 Department of Biochemistry, Faculty of Medicine, Aswan University, Tingar, Egypt; 8 Department of Pharmacology, Faculty of Medicine, Assiut University, Assiut, 71526 Egypt; 9 Basic Medical Science Department, Badr University in Assiut, Assiut, 71526 Egypt

**Keywords:** Gentamicin, ARF, Nanoparticles, LS, IL-6, Caspase 3, Cell biology, Drug discovery, Molecular biology, Diseases, Pathogenesis

## Abstract

Acute renal failure (ARF) is a sudden, significant, and often reversible decline in kidney function, with 25% of all hospital-administered pharmaceuticals potentially causing nephrotoxicity. The study investigates the effectiveness of a novel nanoparticle (NP) formulation of glutathione (GSH) and *Lepidium sativum* (LS) in improving therapeutic outcomes in a rat model of ARF. Sixty adult male albino rats were allocated into ten groups, comprising six rats each, for the study. ARF was created by daily gentamicin (GN) administration for seven consecutive days and various treatment protocols, including chitosan (CS) NPs, spanlastics NPs, as well as conventional, NP formulations of GSH, LS, and their respective combinations. The effect was evaluated through various tests, and properties of nanoparticles were confirmed through characterization processes. The NP compositions markedly enhanced renal function, as seen by reduced urine concentrations of albumin and glucose. Furthermore, the serum concentrations of creatinine (SCr), blood urea nitrogen (BUN), and cystatin C were decreased. Tissue concentrations of nitrite, superoxide dismutase (SOD), and malondialdehyde (MDA), as markers of oxidative stress, were enhanced by both conventional and NP formulations. Additionally, they decreased inflammatory markers such as kidney injury molecule-1 (KIM-1), neutrophil gelatinase-associated lipocalin (NGAL), tumor necrosis factor alpha (TNF-α), and interleukin-6 (IL-6). Histological analysis and immunohistochemical testing revealed that the combination therapy, particularly with the nanoforms, significantly decreased caspase 3 cellular immunoexpression, a sign of kidney cellular damage. The findings show that the ARF renal damage is considerably reduced when NPs containing GSH and LS are administered together. The study suggests a promising pharmacological approach for enhancing kidney regeneration and preserving renal function, potentially aiding in new therapeutic interventions for ARF treatment.

## Introduction

The kidney’s sensitivity to xenobiotics and role in maintaining bodily homeostasis are crucial^1^. Acute renal injury, also known as acute renal failure (ARF), is characterized by rapid decline in kidney function^2,3^. One-fourth of hospital drugs are nephrotoxic, and 65% of ARF patients die in the ICU^4^. Nephrotoxic pathological changes are often caused by renal tubulointerstitial damage^5^.

Gentamicin (GN) is a popular aminoglycoside due to its affordability, minimal resistance, and bactericidal efficacy against gram-negative bacteria^6^. However, it can cause kidney damage, leading to impaired renal function, increased BUN and SCr levels, reduced glomerular filtration, and histopathological changes like mesangial cell hypercellularity, glomerular atrophy, tubular necrosis, degeneration, and vacuolization^7,8^.

The cause of GN-induced ARF in kidneys is a complex process involving oxidative stress, inflammation, and apoptosis^9^. GN binding to phospholipids leads to phospholipidosis, oxidative stress, inflammation, and necrosis, compromising membrane integrity^8,10^. Renal cortical mitochondria generate reactive oxygen and reactive nitrogen species, leading to apoptosis and upregulation of nuclear factor kappa-b (NF-κB) and pro-inflammatory cytokines^11^.

The clinical setting and available resources influence ARF treatment. Over the past decade, evidence supporting various interventions, particularly when combined, has grown, suggesting medicinal herbs as a natural source of antioxidants, anti-inflammatory, and anti-apoptotic properties to reduce kidney oxidative stress and inflammation^12,13^. *Lepidium sativum* (LS), a member of the Brassicaceae family, is an edible annual herb that is grown in Arabia, Europe, India, and the United States. It goes by several names, including Elrshad, pepper cress, and Asaliyo^14^. Analgesic, antipyretic, anti-inflammatory, antimicrobial, anti-diabetic, anti-cancer, analgesic, and diuretic are some of the many traditional medicinal uses of LS^15^. LS seeds have been found to be safe and effective in treating fractures, providing protective benefits to the central nervous system, gastrointestinal tract, respiratory system, and cardiovascular system^16,17^.

Nearly every cell in the body contains glutathione (GSH)^18^, and it is widely distributed in many vital organs and tissues. By scavenging free radicals^19^, antioxidation, detoxification, anti-aging, immune-boosting, and anti-tumor action are all physiological functions of GSH^11^. Consequently, dysregulation of GSH production is seen in a number of medical conditions, such as renal failure^20^, liver injury^21^, diabetes, neurological diseases^22^, organ fibrosis^23^, and cardiovascular disease^24^.

Glutathione and LS orally have several drawbacks, including low bioavailability, easy oxidation, poor stability, and inability to pass through cell membranes, reducing their therapeutic efficacy in various illnesses^11^. Additionally, their metabolism can lead to inactivation and loss of biological activity, making their direct use limited^25^. Researchers are exploring nanotechnology-based medication delivery systems to improve renal treatment, addressing traditional drawbacks like stability, solubility, and toxic effects, making NPs a promising tool for disease diagnosis and treatment^26^.

Nanoparticles are characterized by a very small particle size with a high surface area which enables it reaching or targeting the tissues and enhance cellular uptake. In addition, it composed of bilayer lipid structure and nonionic surfactant that providing prolonged sustained release profile of different drug, spanlastics vesicles act as depot or reservoir of drug and release the drug gradually. Protection against degradation is related to the entrapment of the drug inside core of vesicle which provides protection till reaching site of absorption^27,28^.

In 2011, Kakkar and Kaur introduced spanlastics, a non-immunogenic, biodegradable system that delivers medications via various routes, including oral, topical, nasal, and transdermal methods^29^. Spanlastics’ lipid bilayer structure enables the entry of hydrophilic or lipophilic active substances into biological tissue membranes^30^. This makes spanlastics a promising nanovehicle for encapsulating LS extract, improving its biological activity and oral bioavailability. Particularly for the kidneys, the polymer chitosan (CS) is both biocompatible and biodegradable^31^. Prior studies investigated the potential of CS NPs as oral medication delivery vehicles for renal disease^32^.

In order to evaluate the potential renoprotective advantages of both conventional and NP formulations of LS and GSH as novel therapeutic agents for reducing GN-induced nephrotoxicity in rats, this study was conducted. This study also tracked the function of inflammation, apoptosis, oxidative stress, and nitrosative stress in mediating the effects of our treatment regimens.

## Materials and methods

### Chemicals and reagents

Span^®^60 (sorbitan monostearate), sodium deoxycholate (SDC), and dialysis membrane (molecular weight 12,000–14,000 Da cutoff) were purchased from Sigma (St. Louis, MO, USA). Brij^®^35 (polyethylene lauryl ether) and Myrj^®^52 (polyoxyethylene stearate) were obtained from Merk Co., Germany; Cremophor^®^RH40 (EMAROL H40) was supplied by CISME-Italy. Tween^®^80 and absolute ethanol (99.9%) were supplied by EL-NASR Pharmaceutical Chemicals (CAIRO, EGYPT).

### Gathering of plant parts and extraction

This study’s LS was sourced from a local market in Assiut, Egypt, and then examined by a medicinal plant expert from the Processing and Extraction Unit of Medicinal Plants at Assiut University in Egypt. The LS seeds were gathered all through winter. Following our earlier research, the extraction was carried out^33^.

### Production of *Lepidium sativum* aqueous extract

*Lepidium sativum* aqueous extract was prepared according to previous work with little changes^34^. In a nutshell, 50 g of LS seeds were immersed in 500 ml of warm distilled water and then left on a magnetic stirrer for 5 h at 75 °C and 2000 rpm. After adding the water, the mixture is left to incubate at room temperature for a week. Centrifugation at 1000 rpm was used to separate and purify the generated solution. The final concentrated extract was placed in a place away from light and humidity.

### Analysis of *Lepidium sativum* aqueous extract spectrophotometrically

The LS extract was weighed, dissolved in ethanol, and diluted to concentrations of 5, 10, 15, 20, 25, 30, 35, 40, 45, 50, and 100 μg/mL. Then, the screening absorbance of these concentrations was measured by UV–Vis Spectrophotometer (Lisco, Bargteheide, Germany) at UV wavelengths ranging from 200 to 800 to determine the wavelength of λmax for LS. The maximum absorbance λmax was obtained at 290 nm, and then the calibration curve was generated to examine further characterizations.

### Fabrication of *Lepidium sativum* extract loaded nanospanlastics

The development of LS nanospanlastics was carried out by using the ethanol injection approach based on the previous method described by Kakkar and Kaur^29^. The system is composed of organic and aqueous phases. The organic phase comprises Span 60 and LS dissolved in a little bit of absolute ethanol and stirred at 1000 rpm and a temperature of 70 °C until a clear solution is obtained. The tested edge activators were added to distilled water (10 ml) and heated at the same temperature (70 °C) to develop an aqueous phase. Subsequently, ethanol is slowly evaporated by slowly injecting the organic phase into the water solution using a syringe. The mixture is then continuously stirred with a magnetic stirrer set at 1000 rpm at the same heating temperature for 30 min. Then, the developed LS nanospanlastics dispersions were stored for 24 h at 4 °C in the refrigerator for further measurements. The effect of using different edge activators was studied, and the ratio of Span60 and Edge activators is 2:1. In Table 1, the ingredients for the different formulas were shown.

**Table 1:** Composition and characterization of the developed formulations of *Lepidium sativum* loaded nanospanlastics containing Span 60: Edge activators ratio of (2:1).

Formulation	Extract mg	Span^®^60	Edge activator (100 mg)	Particle size (nm)	PDI	Zeta-potential (mV)	Encapsulation efficiency %
F1	30	200	Tween 80	210 ± 10.4*	0.24 ± 0.2	− 34.9 ± 0.6*	95.5 ± 1.2*
F2	30	200	SDC	324 ± 8.9	0.35 ± 0.4	− 18.1 ± 2.5	90.2 ± 2.5
F3	30	200	Myrj^®^52	270 ± 14.6	0.43 ± 0.6	− 13.2 ± 1.1	88.4 ± 1.3
F4	30	200	Brij^®^35	235 ± 13.5	0.31 ± 0.1	− 9.5 ± 2.2	89.4 ± 1.9
F5	30	200	Cremophor^®^RH40	480 ± 14.6	0.51 ± 0.1	− 19.7 ± 1.2	92.1 ± 0.9

All results are expressed as the mean ± SD (n = 3). *SDC* Sodium deoxy cholate.

*Significant difference (p < 0.05).

### In-vitro characterization of LS-loaded nanospanlastics

#### Assessment of encapsulation efficiency percentage (EE%)

The produced nanospanlastics were tested for LS encapsulation using an indirect approach. Using a benchtop cooling centrifuge set at 4 °C and 20,000 rpm for 2 h, the unloaded LS was isolated from the trapped quantity (Centurion Scientific Ltd., West, UK). Then the content of LS in the filtrate was analyzed by UV–Vis spectrophotometer at a maximum wavelength of extract (λmax of 290 nm). The encapsulation efficiency (EE%) was estimated using the following Eq. (1):

1 $$= \, \left( {{\text{Total initial amount of LS }} - {\text{ amount of LS in filtrate}}} \right)/\left( {\text{Total initial amount of LSE}} \right) \, \times { 1}00$$

#### Assessment of vesicular size, polydispersity index (PDI), and Zeta-potential measurements

A dynamic light scattering approach was utilized to measure vesicle size, PDI, and zeta potential of the developed LS-loaded nanospanlastics by Zetasizer device (Malvern ZS, Ltd., Malvern, UK). The formulations were diluted with an adequate amount of distilled water at room temperature to offer appropriate scattering before measurements. Zeta potential measurements were done by injection of diluted samples into specified cells. All the measurements were carried out in triplicate. Observations were presented as the mean values plus or minus the standard deviation^30^.

#### Assessment of the morphology of LS loaded nanospanlastics

A transmission electron microscope (TEM) (JEOL 100 CX II, Tokyo, Japan) was used to examine the morphology of the chosen LS nanoformulation. To get the right magnification, a high-voltage electron beam was employed. Before imaging, a drop of the tested dispersion was applied to a carbon-coated copper grid, colored with phosphotungstic acid, and allowed to dry in the air.

#### Fourier transform infrared spectroscopy (FTIR) analyses

Tested samples of pure LS extract, plain nanospanlastics, and selected formulations of LS-loaded nanospanlastics were analyzed using an FTIR spectrophotometer (Hitachi-Tokyo-Japan) using the potassium bromide disc method. 5 mg of each sample was mixed with KBr, and then the blend was compressed to form KBr discs using a hydraulic press. All the samples were scanned in the range of 4000–400 cm⁻^1^^35^.

#### Powder X-ray diffraction analysis

The patterns of X-ray were investigated for pure Lepidium sativum, plain nanospanlastics F1, and selected LS nanospanlastics (F1). Analysis of dried samples was performed using a Philips instrument (PW-1050, Bragg–Brentano) diffractometer; Cu K α radiation (35 kV, 40 mA, slit 1.5418 Å).

#### In-vitro drug release analysis

The dialysis method was utilized to investigate the in vitro drug release behavior of LS extract from the selected nanospanlastics formulation F1 compared with free LS extract as described in previous studies^36,37^. In brief, 1 ml of both samples was placed on a dialysis semipermeable membrane that had previously been put in phosphate buffer (pH 6.8 for 24 h) and fixed over the lower opening of a glass tube (2 cm diameter). The tubes containing tested formulations were immersed in a beaker containing 100 ml phosphate buffer (pH 6.8). The experiment was adjusted to 37° ± 0.5 °C in a shaking water bath (Daihan Scientific, Seoul, South Korea) running at 50 rpm. Specific volumes (5 ml) of aliquots were removed at predetermined time intervals (0.5, 1, 2, 4, 6, 8, 12, and 24 h). To maintain sink condition, the volume of removed aliquots was replaced by an equal volume of fresh release medium at the same temperature. The cumulative released amounts of LS were measured via UV–Vis spectrophotometer at a maximum absorbance wavelength of λmax 290 nm. The in vitro release of LS from selected nanospanlastics formulations and free LS solution was investigated for comparison. The experiments were carried out in triplicate, and the results were presented as a mean SD.

#### Kinetic analysis of drug release

The rate at which the drugs were released from the tested mixtures was studied using the linear regression analysis method (R^2^). The results were examined in accordance with different models: zero, first-order kinetic models, and Higuchi diffusion. Using Korsmeyer–Peppas models to investigate the mechanism of drug release that is based on exponent (n) value. Where the exponent value is ≤ 0.5, this pertains to Fickian diffusion, whereas values of 0.5 < (n) < 1.0 correspond to non-Fickian diffusion^38^.

### Preparation and characterization of glutathione loaded chitosan nanoparticles dispersion

Development of GSH-loaded CS NPs was carried out using the ionic gelation approach according to a previously reported method with slight changes^2^. A CS solution (2 mg/ml) was prepared by dissolving 2% glacial acetic acid on a magnetic stirrer at 500 rpm at 30 °C for 24 h. GSH was added into the developed CS solution under stirring at the same rate. Then, sodium tripolyphosphate (TPP) solution (0.5 mg/ml) was added slowly (rate: 1 ml/min) with a syringe into CS-GSH solution at a ratio of 2:1 (CS:TPP) with continuous stirring at 500 rpm for 2 h. The NPs dispersion was evaluated, and, according to the Eq. (1), the EE% of GSH into CS NPs was calculated. Further, particle size, PDI, and potential were assessed according to the previously mentioned method in this text. Then, the dispersion was centrifuged, the filtrate was removed, and the remaining residue was dried at 45 °C. The developed dried CS NPs were stored in a desiccator for further studies.

### Physical stability studies

The stability of the selected nanospanlstics formulation F1 and GSH-loaded CS NPs was analyzed based on previously reported work^38^. The samples tested were placed at 4 °C and 25 °C in a closed vial (20 ml). Measurements of vesicle size, PDI, zeta potential, and encapsulation efficiency % were determined at definite intervals (fresh preparation time, 60 days storage time). All the measurements were carried out three times.

### Experimental animals

Male Wistar rats (200–250 g) were obtained from the Animal Research Faculty of Veterinary Medicine at Assiut University. Animals were maintained in standard polypropylene cages under regulated environmental conditions (temperature 22 ± 2 °C, relative humidity 55 ± 5%, 12-h light/dark cycle) with unrestricted access to normal laboratory feed and water ad libitum. All experimental procedures received approval from the Institutional Animal Care and Use Committee (IACUC) of Veterinary Medicine at Assiut University (Approval No. 06/2024/0277) regarding the treatment and handling of animals used in research, as outlined by the National Institutes of Health.

### Acute renal failure induction

Gentamicin sulfate (GN) was administered intraperitoneally at a dose of 100 mg/kg body weight once daily for 7 days in a row, leading to ARF^39^. Gentamicin induces nephrotoxicity by accumulating in the renal cortex, leading to oxidative stress and tubular necrosis.

### Experimental design

After acclimatization, the rats were split into 10 equal groups, with 6 male rats in each. All drugs were administered to all groups for a period of 7 days. Nanoparticles laden with drugs were supplied at 50% of the dosage of conventional formulations, according to our previous studies^40,41^.

Group I (Control): Rats received normal saline for 7 days as an experimental intervention.

Group II: Rats were given carrier-based NPs of chitosan (CS) and were also induced with ARF.

Group III: Rats were administered carrier-based NPs of spanlastic and were also induced with ARF.

Group IV (ARF): Rats received GN (100 mg/kg/day, i.p.) for 7 days, designated as ARF.

Group V (GN + LS): After gentamicin administration, rats received LS (300 mg/kg/day, orally) for 7 days^33^.

Group VI (GN + GSH): After GN administration, rats received GSH (100 mg/kg/day, i.p.) for 7 days^2^.

Group VII (GN + LS + GSH): After GN administration, rats received LS (300 mg/kg/day, orally) and GSH (100 mg/kg/day, i.p.) for 7 days, serving as the conventional combination group.

Group VIII (GN + LS-NP): After gentamicin administration, rats received LS (150 mg/kg/day, orally) for 7 days.

Group IX (GN + GSH-NP): After GN administration, rats received GSH (50 mg/kg/day, i.p.) for 7 days.

Group X (GN + LS-NP + GSH-NP): After GN administration, rats received a combination of GSH-NP (50 mg/kg/day, i.p.) and LS-NP (150 mg/kg/day, orally) for 7 days.

### Sample collection

Individually housed rats in metabolic cages had their urine samples taken every 24 h at the conclusion of the experiment. Tubes containing urine were preserved with a few drops of 1% sodium azide and kept at – 80 °C until analysis. Prior to scarification, at the end of the study, blood samples were taken and allowed to clot at room temperature and centrifuged at 3000 rpm for 15 min (Model Heraeus Labofuge 400, Thermo Fisher Scientific, USA) to separate serum, which was stored at – 80 °C. We put the rats to sleep by inhaling 5% isoflurane before euthanasia. We swiftly terminated the rats’ lives by causing neck dislocation when they failed to react to head and limb stimulation. Rats were considered dead after 10 s of cervical dislocation if they ceased breathing and did not respond to systemic stimulation. Kidneys were excised, washed in ice-cold isotonic saline, blotted dry, weighed, divided, and homogenized in ice-cold phosphate-buffered saline (PBS, pH 7.4) using a tissue homogenizer (Model T25 digital ULTRA-TURRAX^®^, IKA^®^ Works, Germany). The homogenates were centrifuged at 10,000 rpm for 20 min at 4 °C, and the supernatants were collected and stored at – 80 °C for biochemical analyses^42^. It was necessary to preserve kidney tissues in 10% phosphate-buffered formalin for histopathological and immunohistochemical analyses before proceeding.

### Biochemical analyses

#### Measurement of urinary albumin parameter

Urinary albumin levels were measured using a bromocresol green (BCG) dye-binding commercially available assay kit (Schiffgraben, Hannover, Germany)^43^.

#### Assessment of urinary glucose

A commercially available assay kit called glucose oxidase–peroxidase (GOD-POD) was used to assess the urinary glucose levels (Schiffgraben, Hannover, Germany)^44^.

#### Analysis of serum creatinine parameter

Serum creatinine levels were determined using the modified Jaffe’s kinetic technique using a commercially available assay kit (Schiffgraben, Hannover, Germany)^45^.

#### Examination of serum urea parameter

Serum urea concentration was determined using the urease-glutamate dehydrogenase (GLDH) enzymatic technique using a commercially available kit (Schiffgraben, Hannover, Germany)^46^.

#### Measurement of serum cystatin C parameter

A commercial ELISA kit (ERCST3, Thermo Fisher) was used to quantify serum cystatin C levels according to the manufacturer’s protocol^47^.

#### Exploration of serum nitrite levels

Nitrite levels, an indication of nitric oxide production, were evaluated using the Griess reagent assay^48^.

### Oxidative stress markers in kidney tissue

#### Superoxide dismutase (SOD) activity measurement

The ability of the SOD to inhibit the xanthine-xanthine oxidase system’s superoxide radical production and its subsequent oxidation of nitroblue tetrazolium (NBT) was measured using a commercially available spectrophotometric kit^49^.

#### Malondialdehyde (MDA) content measurement

The MDA levels, a marker of lipid peroxidation, were measured using the thiobarbituric acid (TBA) assay^40,50^.

### Measurement of kidney injury markers

The KIM-1 levels in kidney tissue were tested using a rat-specific sandwich ELISA kit purchased from Shanghai, Hangzhou, Zhejiang, China, Sunlong Biotechnology^41,42,51^.

### Inflammatory cytokine assessments

The TNF-α levels in kidney tissue homogenates were evaluated using a high-sensitivity rat TNF-α ELISA kit purchased from Shanghai, Hangzhou, Zhejiang, China, Sunlong Biotechnology^52^.

### Histopathological examination

Kidney tissues were fixed in 10% neutral buffered formalin for 24 h, dehydrated via graded alcohols, cleaned in xylene, and embedded in paraffin. Sections of 5 μm thickness were cut and placed on glass slides. The sections were deparaffinized, rehydrated, stained, washed, differentiated, blued, counterstained, dehydrated, cleared, mounted, and histological alterations were evaluated under a light microscope at different magnifications. The semiquantitative evaluation of tubular degeneration (D), necrosis (N), tubule interstitial nephritis (IN), and overall histological scores ranged from 0 to 4 according to previous study^53^.

Acidophilus-containing vacuolization and stained bodies of different sizes were regarded as D in the cytoplasm of the proximal tubule epithelial cells. Tubular degeneration scoring: 0 D; no degeneration; 1 Mild D: a little, sparsely focused D directly beneath the capsule (0%–10); 2 Moderate D: along the tubular section (10%–25) and for a few focal focus D; 3 Diffuse and considerable D along the tubular segment (% 25–50) is considered severe D. 4 Extremely severe D: degeneration exceeded 50%.

Acellular portions of tubules, dark acidophilic cytoplasm, loss of tubular epithelial cells into the tubular lumen, and loss of nucleus epithelial cells are all signs of tubular necrosis (N). 0 Lack of N; 1 Mild N: a tiny amount of focused N just below the capsule (0%–10); 2 Moderate N: along the tubular section (10%–25) and for a few focal focus N; 3 The tubular segment has diffused and substantial N (% 25–50) in severe D; 4 Extremely severe N: N exceeded 50%.

The infiltration of inflammatory cells into interstitial and perivascular spaces is known as tubulointerstitial inflammation (IN). Score: IN absent; 0 and Mild IN: a few infiltration fragments centered on the perivascular region (0–5%); 1 & Moderate IN: often infiltrations including numerous focal regions and the cerebral interstitial space (5–10%); 2 and Severe IN: regions of diffuse and substantial infiltration (15–25%); 3 and Extremely severe IN: IN exceeded 50%; 4.

A total histologic score (HS) is calculated as follows: D/2 + N + IN/2. According to the scoring system, a score of 0–2 indicates normal HS, 2–5 indicates mild HS, 5–8 indicates moderate HS, and 8 and above indicate severe HS.

### Immunohistochemistry for Caspase-3

Immunohistochemical analysis was performed to detect caspase-3 expression in renal tissues. The study involved deparaffinizing kidney sections, retrieving antigens, blocking endogenous peroxidase activity, incubating sections with rabbit anti-caspase-3 primary antibody, biotinylated secondary antibody, and streptavidin–horseradish peroxidase conjugate. Immunoreactivity was visualized using DAB chromogen solution, and sections were prepared. Negative controls were processed by omitting the primary antibody. The expression of caspase-3 was evaluated by counting positively stained cells in ten random high-power fields (400 × magnification) for each section. Positive cell percentages as a proportion of total cell count were used to express these values, and the mean ± SE for each group was calculated after that. The intensity of staining and the percentage of positive cells were assessed semi quantitatively. Images analysis was done using ImageJ, a professional image analysis program^33,54^.

### Statistical analysis

Using the Shapiro–Wilk test, we determined that all the parameters under consideration had normal distributions; the results showed that p > 0.05. After running the data via one-way ANOVA, we used Tukey’s multiple comparison test to see if there was any statistical significance. A statistically significant result was defined as p < 0.05. The data is summarized as the means plus or minus the standard error of the mean (SEM). We used GraphPad Prism^®^ (version 8) to do the statistical analyses.

## Results

### Development of *Lepidium sativum* loaded nanospanlastics

Table 1 presents the composition and characterization of the developed LS nanospanlastics.

### In-vitro characterization of nanospanlastics formulations

The measurements of vesicular size of the developed formulations showed the sizes ranged from 210 ± 10.4 to 480 ± 14.6 nm (Table 1). The value of PDI of the fabricated preparations ranged from 0.24 ± 0.15 to 0.51 ± 0.12. Further, Table 1 shows that Zeta-potential values were negative and ranged from − 9.5 ± 2.2 to − 28.9 ± 1.60 mV. The observed high values of zetapotential confirm the colloidal stability of the developed nanovesicle dispersions. Regarding to the percentage of EE %, the results showed that all the formulations have high encapsulation % ranged from 88.4 ± 1.3 to 95.5 ± 1.2%.

According to aforementioned results (Table 1) the formulation (F1) which contains Span 60 (200) and Tween 80 (100) as edge activator (2:1) exhibited significant (p < 0.05) higher value EE% (95.5%), smaller measurement of vesicle size, suitable value of PDI and significant (p < 0.05) higher value of zetapotential (− 34.9 ± 0.60) (Fig. 2) compared with other examined edge activators. Thus, LS loaded nanospanlastics (F1) was selected for further investigations.

#### Assessment of the morphology of *Lepidium sativum* loaded nanospanlastics by transmission electron microscopy

**Fig. 1:**
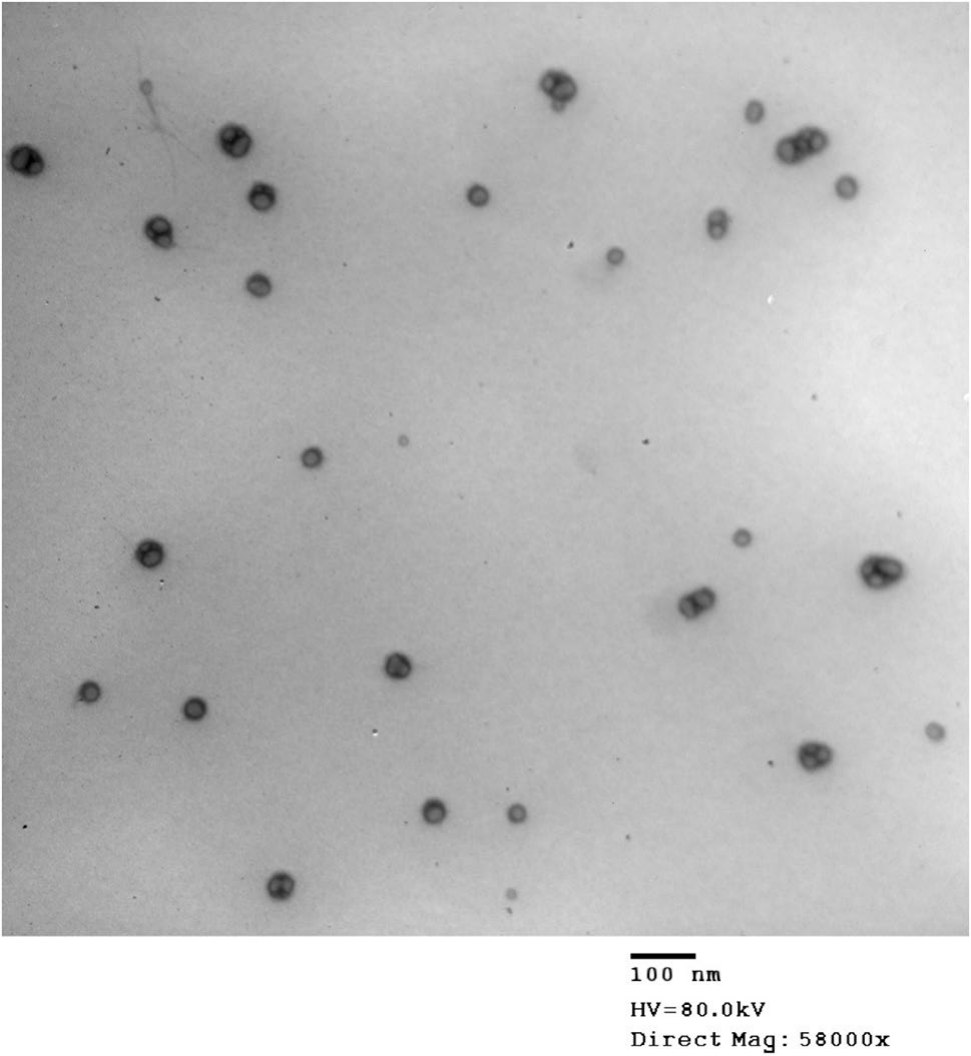
TEM image of *Lepidium sativum* loaded nanospsnlastics (F1).

**Fig. 2:**
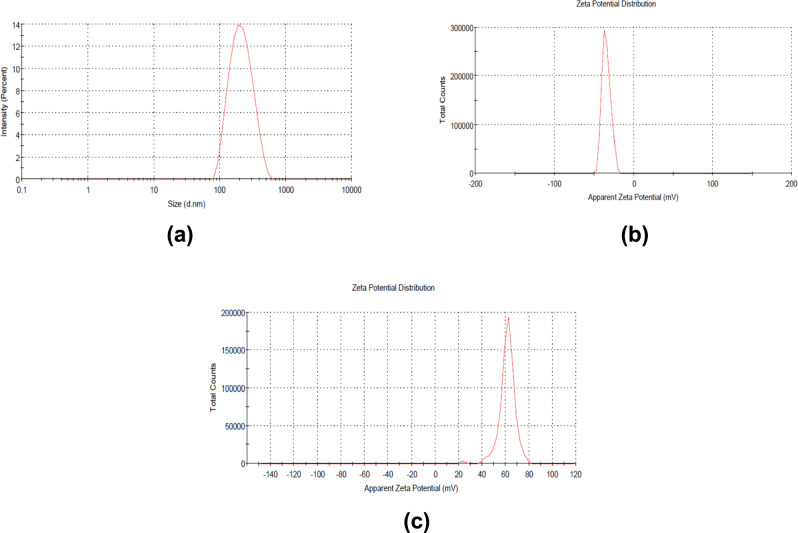
Characterization of developed nanosystems (**a**) size distribution curve, (**b**) zeta potential of *Lepidium sativum* nanospanlastics (F1) and (**c**) zeta potential of glutathione chitosan nanoparticles.

The morphology of the developed nanospanlastics F1 was depicted in Fig. 1. The nanovesicles were found homogenous, uniform distributed in size (Fig. 2), spherical and showed no aggregation.

#### Fourier transform infrared spectroscopy analysis

**Fig. 3:**
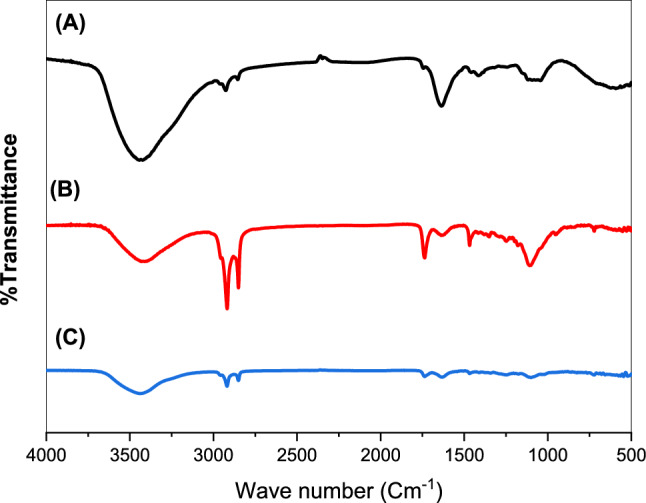
FTIR spectra of (**A**) Free *Lepidium sativum*, (**B**) plain nanospanlstics and (**C**) *Lepidium sativum* nanospanlastics (F1).

As shown in (Fig. 3A)**,** FTIR spectrum of Lepidium sativum presented a distinctive band at 3450, for O–H stretching vibrations, small peaks were at 2924 cm^−1^ and 2800 due to C-H vibrations**.** The characteristic peaks from1400–1600 cm^−1^ were assigned to COO-stretching vibrations and amide groups stretching vibrations. Also, the band that appeared at 1150 cm^−1^ because of vibrations of C–O, C–O–C glycosidic and C–O–H vibrations**.**

The spectrum curve (B) of Plain F1 depicts bands at 3400 cm^−1^ for –OH stretching and at 2900 and 2840 cm^−1^ For C–H stretching. Also, the spectrum shows a peak at 1735 cm^−1^ 1730 cm^−1^ that reflecting 5-membred cyclic ring structure and C=O ester stretch, respectively These mentioned peaks may be considered the characteristic peaks of Span 60 and Tween 80. The spectrum of selected formulation F1 revealed shifting combined with changing intensity (1400–1600 cm^−1^) and the disappearance of characteristic observed peaks (1150 cm^−1^) of LS.

#### Powder X-ray diffraction analysis

**Fig. 4:**
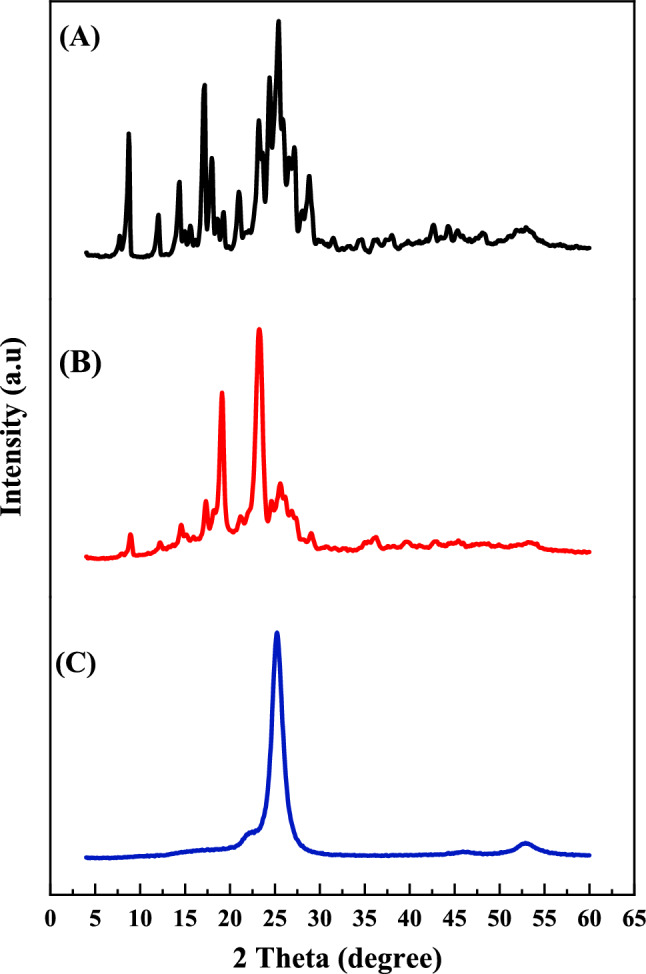
X ray diffraction of (**A**) Free phytochemical (*Lepidium sativum*), (**B**) Physical mixture of spanlastics ingredients and (**C**) selected *Lipidium sativum* nanospanlastics (F1).

The patterns were illustrated in Fig. 4A–C. As shown in the diffractogram (Fig. 4A), pure LS showed multiple sharp intense peaks around 20° 2θ values which reveals its crystalline character. While the diffraction pattern of selected nanospanlastics (F1) (Fig. 4C) confirms an amorphous nature of the compound due to the disappearance of sharp intense peaks of LS. It was noticed in pattern (B) of physical mixture, some of crystallinity peaks were still present in the structure.

#### In-vitro drug release and kinetic analysis

**Fig. 5:**
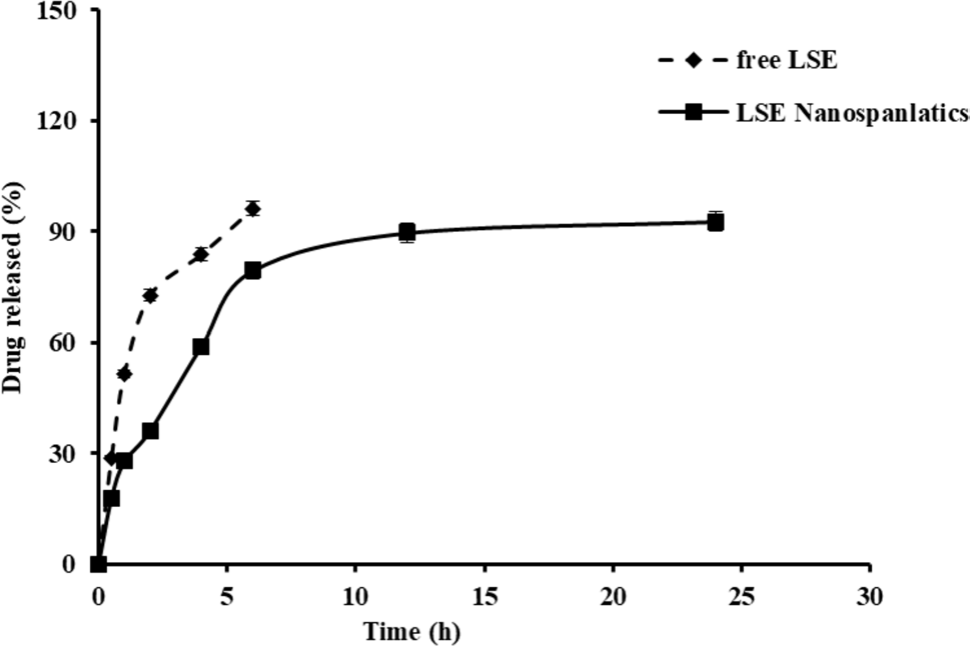
Cumulative *in-vitro* release profiles of *Lepidium sativum* from nanospanlastics dispersion F1 and free drug solution in phosphate buffer pH 6.8 at 37 °C. Data are presented as mean ± SD (n = 3). The experiment was conducted in triplicate, and the data are shown as a mean ± standard deviation (SD).

Figure 5 presents the release patterns of LS from nanospanlastics formulation F1 and solution of LS pure extract (control) for 24 h. The results revealed that selected formulation F1 exhibited significant (P < 0.05) slower rate of drug release as compared with free extract solution (all the active drug is released in the first 4 h). Also, the release manner of examined nanospanlastics formulation displayed an initial burst drug release which lasted to 2 h of 36%, the rapid release is followed by sustained pattern of drug release that up to 24 h of 92.7%. The results of drug release from F1 were analyzed by linear regression of different mathematical models, the calculated correlation coefficient value R^2^ revealed that the drug release was best fitted to Higuchi model (R^2^ = 0.992). Korreysmyr Peppas equation was applied to confirm the mechanism of drug release from nanovesicles, the calculated release exponent n value was found to be 0.55.

### Glutathione chitosan nanoparticles

According to the dynamic light scattering approach the aqueous dispersion exhibited small particle size of 321 ± 7.8 nm, small value of PDI (0.350 ± 0.23) and high value of zetapotential (61.5 ± 1.6) (Fig. 2). Also, the formulating had an acceptable value EE % of 85 ± 2.32%,

### Stability study

Table 2 shows physicochemical properties of selected formulation F1 and GSH-CS NPs following 60 days of storage at a temperature of 4.0 and 25.0 °C, no settling or changes in the visual appearance were noticed. Besides, the examined samples showed non-significant (p > 0.05) alterations in vesicle size, PDI and percentage of encapsulation of the stored formulations. These results suggested that nanovesicles (spanlastics) might offer a protective vehicle of the used phytochemical *Lepidium sativum*, maintaining its stability during storage time.

**Table 2:** Physicochemical properties of the prepared nanospanlastics (F 1, *Lepidium sativum* nanospanlastics and Glutathione chitosan nanoparticles) after 60 days storage at 4 °C and 25 °C.

Parameters	*Lepidium Sativum* nnaospanlastics F1			
	Zero time		60 days	
Temperature	4 °C	25 °C	4 °C	25 °C
Visual appearance	No sedimentation	No sedimentation	No sedimentation	No sedimentation
EE%	95.5 ± 1.2	95.5 ± 1.2	93.86 ± 1.4^ns^	93.10 ± 1.9^ns^
PDI	0.2 ± 0.2	0.2 ± 0.1	0.3 ± 0.02	0.3 ± 0.02
Size (nm)	210 ± 10.4	210 ± 10.4	230.11 ± 12.3^ns^	240.41 ± 10.2^ns^
Zeta potential	− 34.9 ± 0.6	− 34.9 ± 0.6	− 31.9 ± 0.3^ns^	− 30.2 ± 0.5ns
	Glutathione chitosan nanoparticles			
Zeta potential	61.5 ± 1.6	61.5 ± 1.6	56.9 ± 2.6	54.1 ± 2.3
PDI	0.4 ± 0.2	0.4 ± 0.2	0.4 ± 0.03	0.4 ± 0.09
Size (nm)	321 ± 7.8	321 ± 7.8	334 ± 9.5	34 ± 11.2

Results presented as a mean value ± S.D. (n = 3).

Non significance (p > 0.05).

### *Lepidium sativum*, glutathione, and their nanoparticle formulations’ impacts on glucose and urine albumin concentrations in rats that had ARF induced

**Fig. 6:**
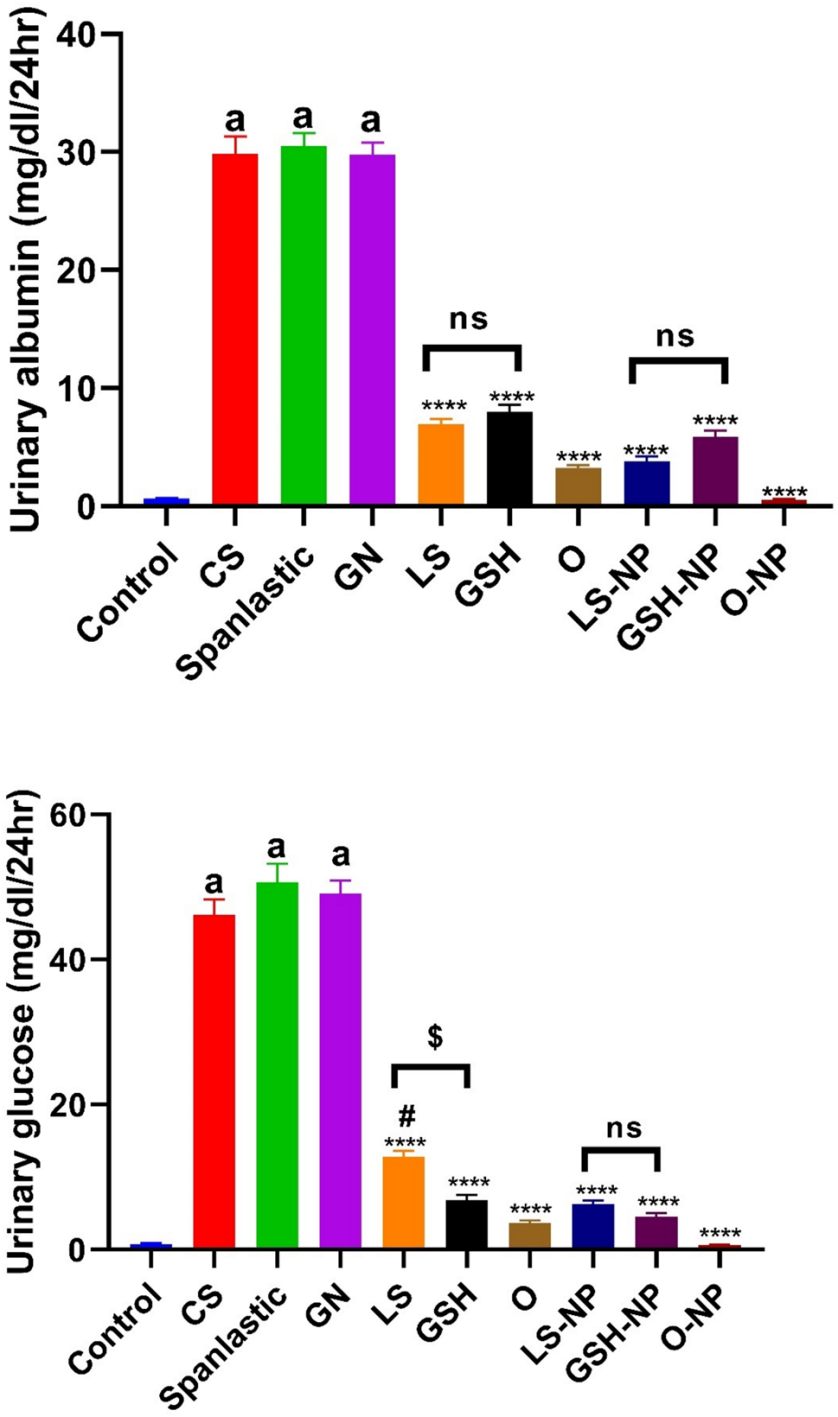
Urine albumin and glucose levels in rats with gentamicin (GN)-induced acute renal failure (ARF) as a function of conventional, nanoparticle (NP), and combination glutathione (GSH) and *Lepidium sativum* (LS) formulations. This data consists of the means with standard error of the mean (n = 6). ^a^p < 0.0001 in comparison to the control group. ****p < 0.0001 in comparison to the ARF-induced group. ^#^p < 0.05 when compared with the related nanoparticle group. ^$^p < 0.05 when compared LS to GSH group. Non significance (p > 0.05). *CS* chitosan, *O* LS-GSH combination, *GSH-NP* glutathione nanoparticles, *LS-NP* *Lepidium sativum* nanoparticles, *O-NP* LS-GSH nanoparticle combinations.

The GN group had markedly increased urine albumin and glucose concentrations relative to the negative control group (p < 0.0001). Rats administered CS and spanlastics NPs exhibited no notable alterations in urine albumin and glucose concentrations relative to rats with ARF. Furthermore, we compared rats subjected to acute renal failure to those that received either standard treatments or NPs of LS, GSH, or both. The urine albumin and glucose levels were significantly lower in the latter groups (p < 0.0001). The NP formulation of LS, GSH, and their combination caused a significant decrease in rats compared to the usual forms of these compounds (Fig. 6).

Notable disparities were detected in urine albumin levels between the traditional forms of LS and GSH. No variations in glucose levels were seen between these types. Furthermore, an analysis of the nano forms of LS and GSH revealed no significant disparities in urine albumin or glucose levels.

### Blood creatinine, urea, and cystatin C levels in rats that had ARF triggered were examined in relation to *Lepidium sativum*, glutathione, and their nanoparticle formulations

**Fig. 7:**
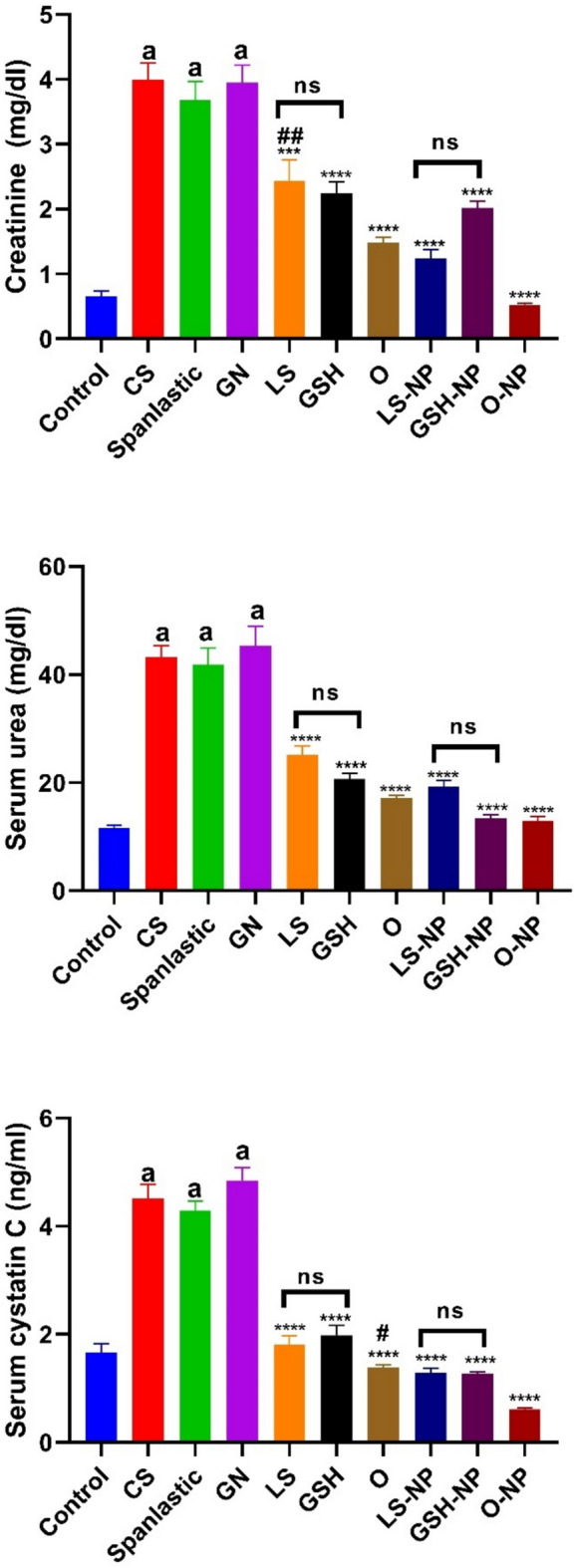
Impact of traditional, nanoparticle, and mixed glutathione (GSH) and *Lepidium sativum* (LS) formulations on creatinine, urea, and cystatin C serum levels in rats afflicted with gentamicin (GN)-induced acute renal failure (ARF). The data shown are the means with standard error of the mean (n = 6). ^a^p < 0.0001 in comparison to the control group. ***p < 0.001 and ****p < 0.0001 in comparison to the ARF-induced group. ^#^p < 0.05 and ^##^p < 0.01 when compared with the related nanoparticle group. Non significance (p > 0.05). *CS* chitosan, *O* LS-GSH combination, *GSH-NP* glutathione nanoparticles, *LS-NP* *Lepidium sativum* nanoparticles, *O-NP* LS-GSH nanoparticle combinations.

Serum levels of BUN, SCr, and cystatin C were significantly higher in the gentamicin group compared to the control group (p < 0.0001). When CS and spanlastics NPs were given to rats, their serum BUN, SCr, and cystatin C levels did not change significantly compared to rats with ARF. Moreover, when rats with ARF were compared to rats that received either standard treatments or NPs of LS, GSH, or both, the levels of BUN, SCr, and cystatin C in their blood were found to be significantly lower (p < 0.0001). The decrease was marked in rats that were given the NPs form of LS, GSH, or both together, compared to the conventional forms of these substances (Fig. 7). No significant differences were seen in serum levels of BUN, creatinine, and cystatin C between the conventional forms of LS and GSH, as well as between the nano forms of LS and GSH.

### *Lepidium sativum*, glutathione, and their nanoparticle formulations’ impacts on tissue malondialdehyde, nitrite and superoxide dismutase levels in rats that had ARF induced

**Fig. 8:**
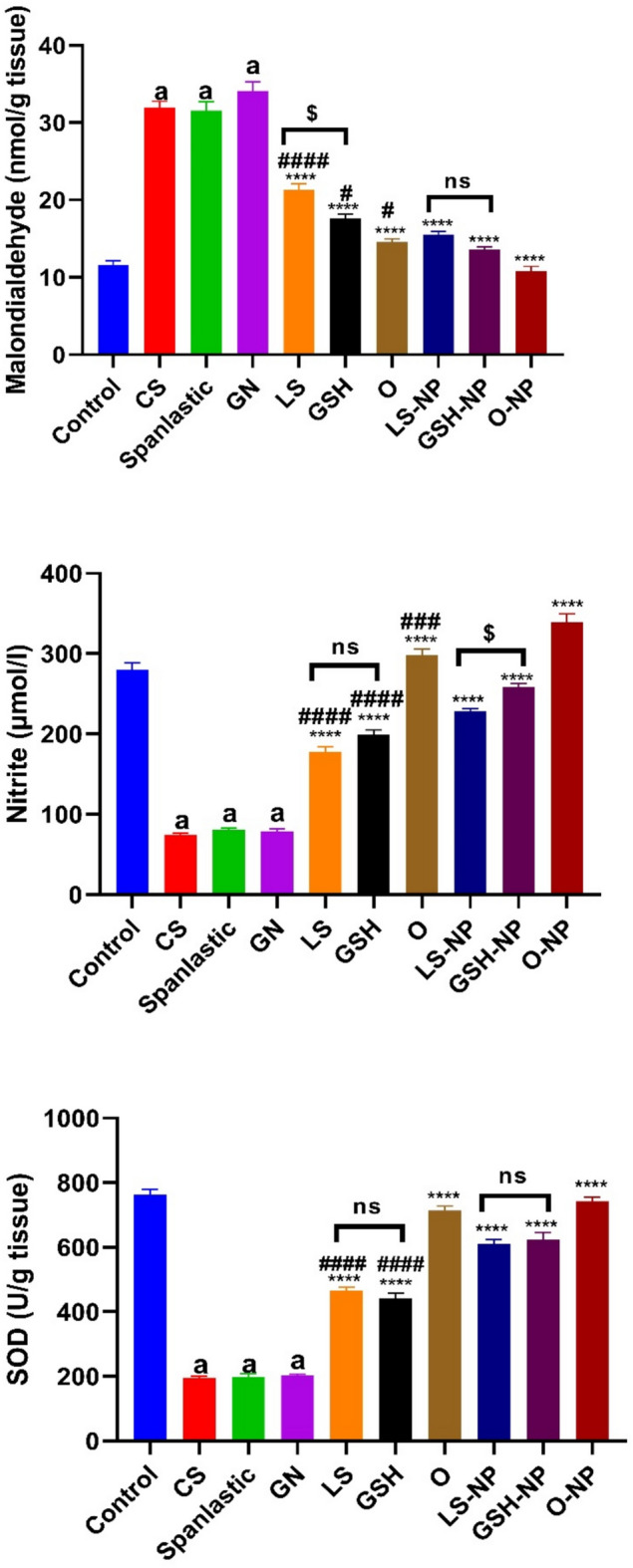
Acute renal failure (ARF) caused by gentamicin (GN) in rats: effects of conventional, nanoparticle (NP), and combination forms of glutathione (GSH) and Lepidium sativum (LS) on tissue levels of malondialdehyde, nitrite, and superoxide dismutase (SOD). The data consists of the means with standard error of the mean (n = 6). ^a^p < 0.0001 in comparison to the control group. ****p < 0.0001 in comparison to the ARF-induced group. ^#^p < 0.05, ^###^p < 0.001 and ^####^p < 0.0001 when compared with the related nanoparticle group. ^$^p < 0.05 when compared LS to GSH group. Non significance (p > 0.05). ^$^p < 0.05 when comparing the LS and LS-NP groups to the GSH and GSH-NP groups, respectively. Non significance (p > 0.05). *CS* chitosan, *O* LS-GSH combination, *GSH-NP* glutathione nanoparticles, *LS-NP* *Lepidium sativum* nanoparticles, *O-NP* LS-GSH nanoparticle combinations.

When rats were given gentamicin, their tissue MDA levels went up a lot and their nitrite and SOD levels went down a lot (p < 0.0001) compared to the negative control group. Rats administered CS and spanlastics exhibited no significant variations in MDA, nitrite, and SOD levels relative to the GN group. Tissue MDA levels dropped significantly when conventional therapies or NP forms of LS, GSH, or a mix of the two were used. Conversely, nitrite and SOD levels exhibited a considerable rise (p < 0.0001) in comparison to the ARF-positive control group. All groups of animals treated with NPs had significantly higher levels of tissue nitrite and SOD in comparison to animals that underwent identical routine treatments (Fig. 8). At the same time, tissue MDA levels were significantly lower.

Notable differences were identified in MDA levels between the conventional forms of LS and GSH, although no variation in nitrite and SOD levels were seen between these kinds. Furthermore, an examination of the nano forms of LS and GSH indicated considerable discrepancies in nitrite levels, but there was no variation in MDA and SOD levels between these nano forms.

### 3.8 Effects of *Lepidium sativum*, glutathione, and a combination of the two on levels of neutrophil gelatinase-associated lipocalin and tissue kidney injury molecule-1 in rats subjected to ARF experimental induction

Tissue KIM-1 and NGAL levels were significantly higher in the GN-treated group of rats compared to the control group (p < 0.0001). Unlike the GN induced ARF group, rats administered CS or spanlastics exhibited no significant alterations in the levels of KIM-1 and NGAL in their tissues.

**Fig. 9:**
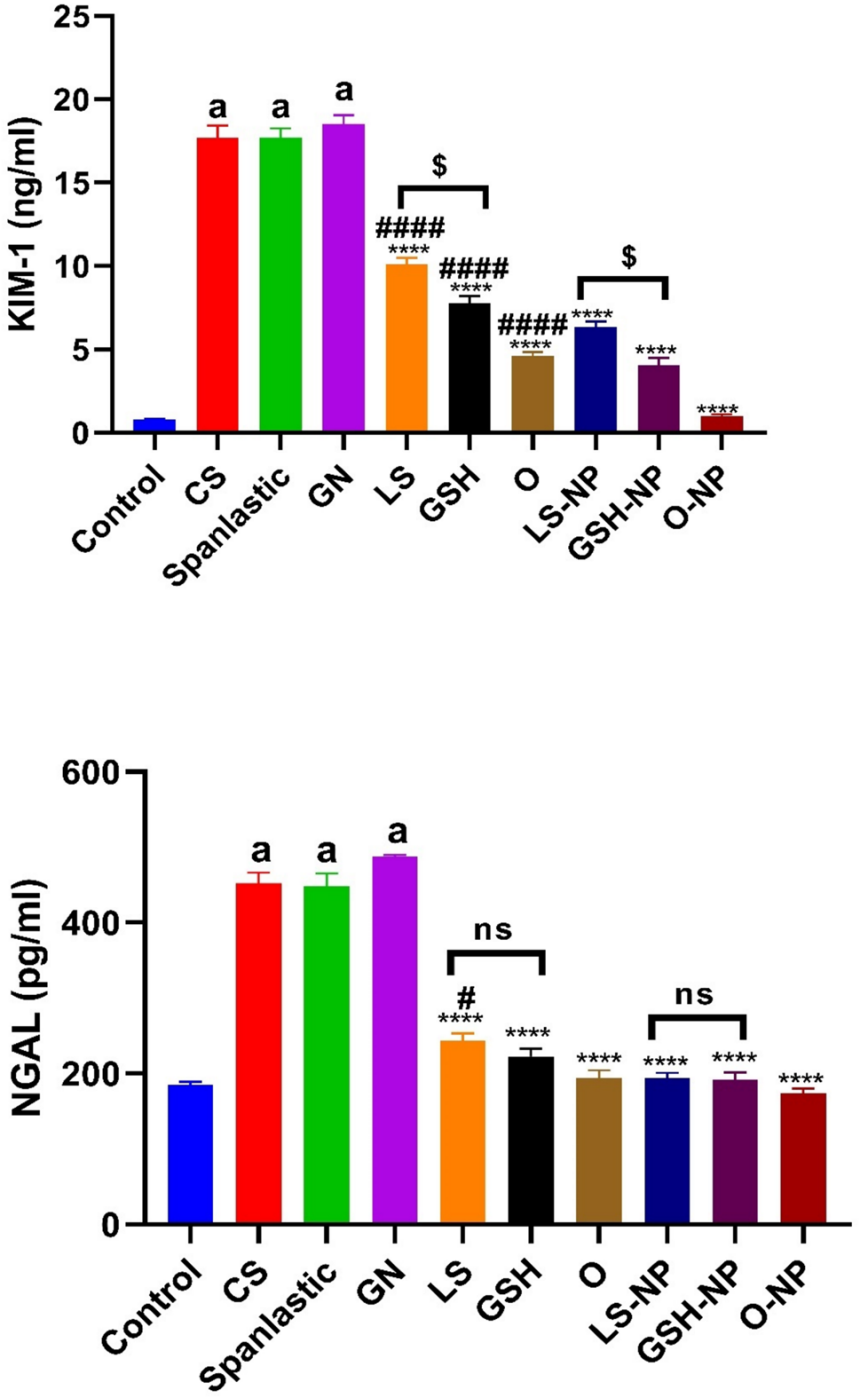
Acute renal failure (ARF) induced by gentamicin (GN) in rats: effects of conventional, nanoparticle (NP), and combination forms of glutathione (GSH) and Lepidium sativum (LS) on tissue levels of neutrophil gelatinase-associated lipocalin (NGAL) and Kidney injury molecule-1 (KIM-1). The data shown are the means with standard error of the mean (n = 6). ^a^p < 0.0001 in comparison to the control group. ****p < 0.0001 in comparison to the ARF-induced group. ^#^p < 0.05 and ^####^p < 0.0001 when compared with the related nanoparticle group. ^$^p < 0.05 when comparing the LS and LS-NP groups to the GSH and GSH-NP groups, respectively. Non significance (p > 0.05). *CS* chitosan, *O* LS-GSH combination, *GSH-NP* glutathione nanoparticles, *LS-NP* *Lepidium sativum* nanoparticles, *O-NP* LS-GSH nanoparticle combinations.

In comparison to the GN-induced ARF group, the conventional and NP formulations of LS, GSH, or both had significantly decreased levels of KIM-1 and NGAL in the tissues (p < 0.0001). Tissue KIM-1 and NGAL levels were significantly lower in rats with NP formulations of LS, GSH, or a mix of the two, as shown in Fig. 9, when compared to rats treated with standard pharmaceuticals.

Notable differences were identified in KIM-1 levels between the conventional forms of LS and GSH, although no variation in NGAL levels were seen between these kinds. Furthermore, an examination of the nano forms of LS and GSH indicated considerable discrepancies in KIM-1 levels, but there was no variation in NGAL levels between these nano forms.

### *Lepidium sativum*, glutathione, and their nanoparticle formulations’ impacts on tissue tumor necrosis factor alpha and interleukin 6 levels in rats that had ARF induced

**Fig. 10:**
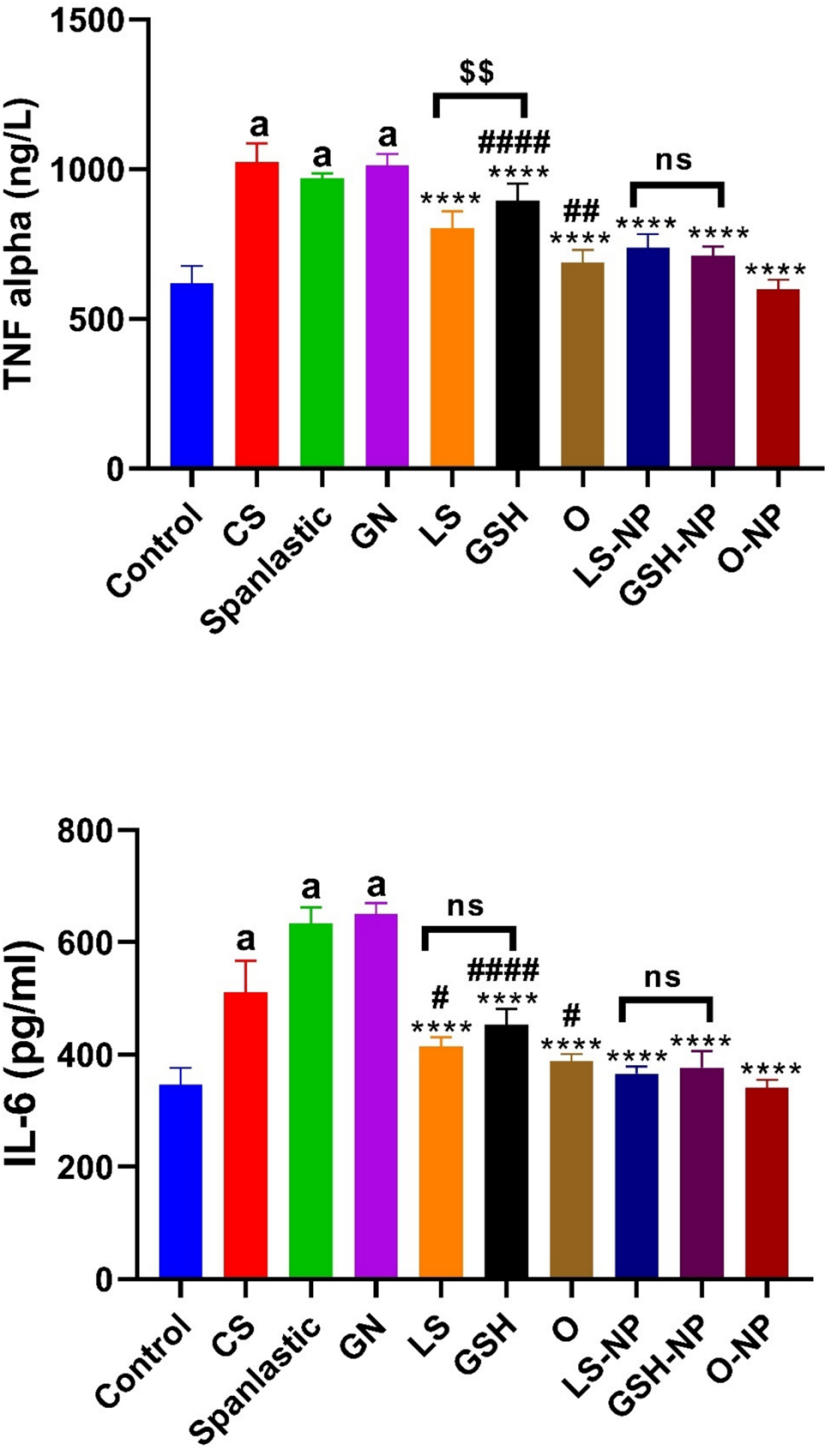
Assessment of the effects of traditional, nanoparticle (NP), and mixed forms of glutathione (GSH) and Lepidium sativum (LS) on tumor necrosis factor alpha (TNF-α) and interleukin-6 (IL-6) serum levels in rats induced acute renal failure (ARF) by gentamicin (GN). This data consists of the means with standard error of the mean (n = 6). ^a^p < 0.0001 in comparison to the control group. ****p < 0.0001 in comparison to the ARF-induced group. ^#^p < 0.05, ^##^p < 0.01 and ^####^p < 0.0001 when compared with the related nanoparticle group. ^$$^p < 0.01 when comparing the LS to the GSH group. Non significance (p > 0.05). *CS* chitosan, *O* LS-GSH combination, *GSH-NP* glutathione nanoparticles, *LS-NP* *Lepidium sativum* nanoparticles, *O-NP* LS-GSH nanoparticle combinations.

Compared to the negative control group, the gentamicin group’s tissue TNF-α and IL-6 levels were significantly higher (p < 0.0001). Rats administered CS and spanlastics NPs showed no discernible change in tissue TNF-α and IL-6 levels when compared to rats given ARF. Furthermore, tissue levels of TNF-α and IL-6 in tissue were significantly lower (p < 0.0001) in rats with ARF than in rats who received either normal treatments, LS, GSH NPs, or both. When rats were administered LS, GSH, or both in NP form instead of the traditional forms, there was a noticeable drop (Fig. 10).

Notable changes were observed in TNF-α levels between the conventional forms of LS and GSH, however no variation in IL-6 levels were seen between these types. Furthermore, an analysis of the nano forms of LS and GSH found no disparities in tissue levels of TNF-α and IL-6 between these nano forms.

### Histopathology evaluation

**Fig. 11:**
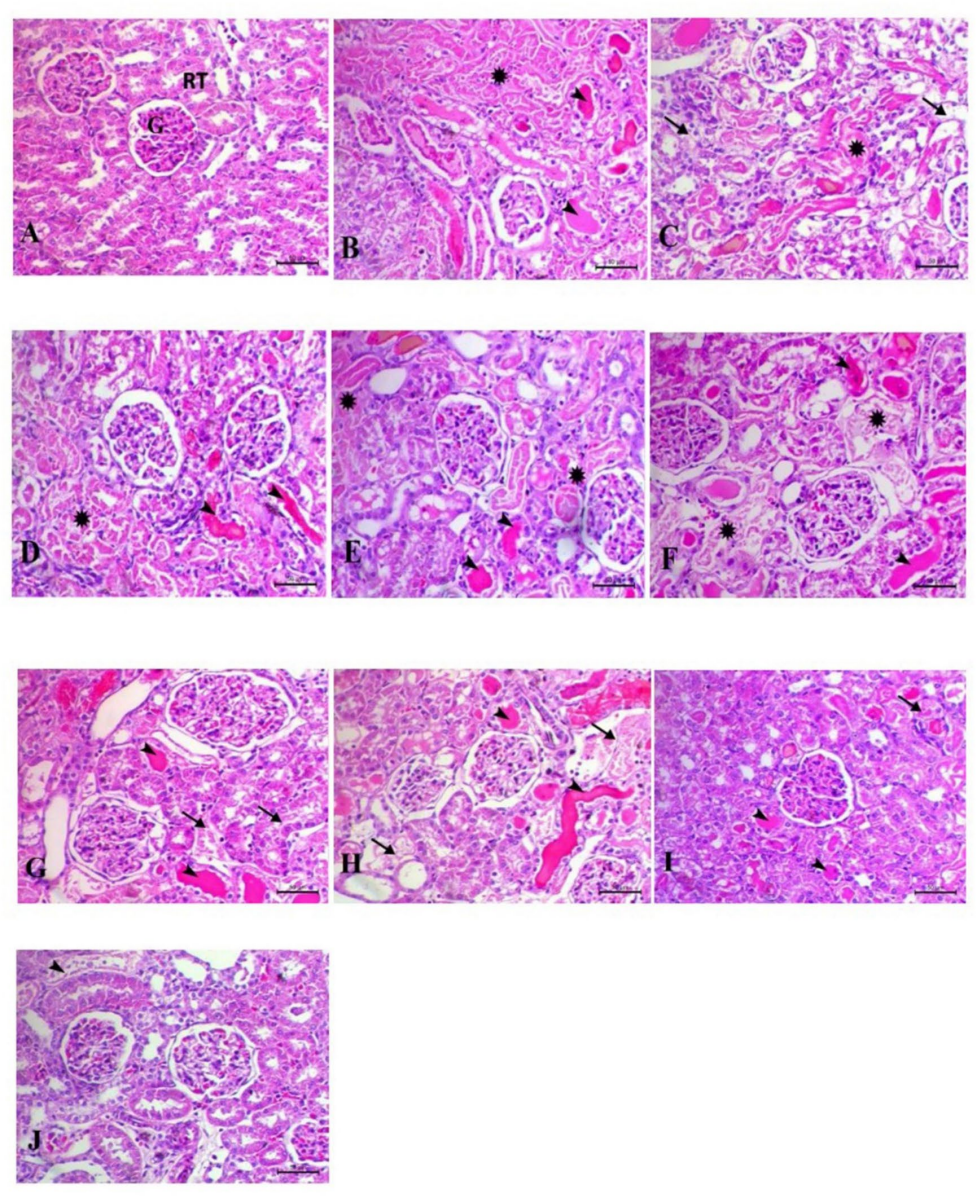
Histochemical staining of rat kidneys in different treatment groups. The bar representing the scale is 50 μm. (**A**) The renal histo-architecture is normal in the control group; the glomerulus (G) and tubules (RT) are also in good shape. On the other hand, the gentamicin (GN) (**B**) group shows massive coagulative tubular necrosis, loss of renal tubule architecture (asterick), and homogeneous eosinophilic protein fluid within the lumen of the kidney tubule (arrowheads). Necrosis of many tubules (arrow) and hydropic degeneration (asterick) in the CS and nanospanlastic groups, with protein fluid within the lumen of the kidney tubule (arrowheads) in sets (**C**) and (**D**). In (**E**) and (**F**), the GSH and LS groups demonstrate reduced tubular necrosis, while astericks indicate hydropic degeneration. Arrowheads indicate the presence of protein fluid within the lumen of the kidney tubule. The tubular architecture shows some improvement in the GSH-NPs and LS-NPs groups, but there are still some spots of tubular necrosis (asterisks) and protein fluid within the lumen of the renal tubule (arrowheads) in the G and H group. The tubular architecture of the kidneys significantly improved in the I and J groups treated with GSH-LS and GSH-LS-NPs, while there were some tubular epithelial degenerations (arrows) and fluid proteins within the lumen of the kidney tubule of certain tubules (arrowheads). *GN* gentamicin, *GSH* glutathione, *LS* *Lepidium sativum*, *NPs* nanoparticles.

**Fig. 12:**
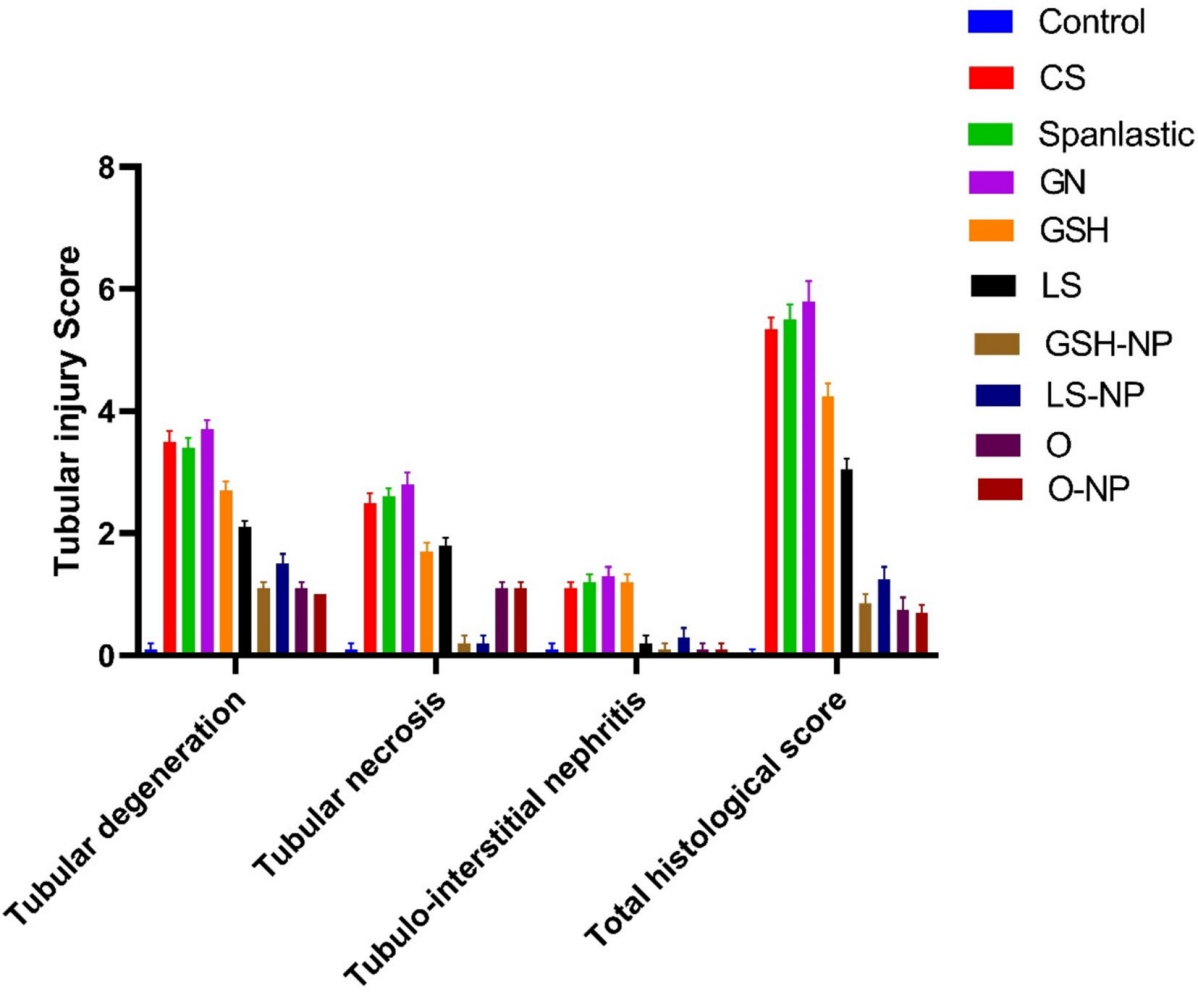
Tubular renal injury score. Pathological analysis using a semiquantitative scoring system with a range of 0 to 4. *GN* gentamicin, *CS* chitosan, *GSH* glutathione, *GSH-NP* glutathione nanoparticles, *LS* *Lepidium sativum*, *LS-NP* *Lepidium sativum* nanoparticles, *O* LS-GSH combination, *O-NP* LS-GSH nanoparticle combinations.

The lesion tubular injury scores from the histopathological data for all groups analyzed are reported in Fig. 12. The assessment of the kidneys in the negative control group demonstrated normal architecture. The GN-induced ARF group exhibited alterations in both tubular and glomerular structures. The tubular alterations exhibited extensive coagulative necrosis accompanied by homogeneous eosinophilic proteinaceous fluid within the renal tubule lumens, alongside significant glomerular abnormalities. The Cs and nanospanlastics treated groups exhibited identical lesions to those observed in the GN-induced acute renal failure group, together with significant hydropic vacuolation of the renal tubular epithelium. GSH, LS, GSH-NPs, and LS-NPs demonstrated minimal enhancement of tubular cells, with partial restoration of renal tubule morphology and reduced protein fluid accumulation within the lumen. The combination of conventional medications and nano-loaded pharmaceuticals demonstrated significant enhancement of tubular cells, resulting in complete restoration of renal tubule architecture, with minimal buildup of protein fluids observed solely in the conventional drugs combination group (Figs. 11, 12).

### Immunohistochemical analysis

**Fig. 13:**
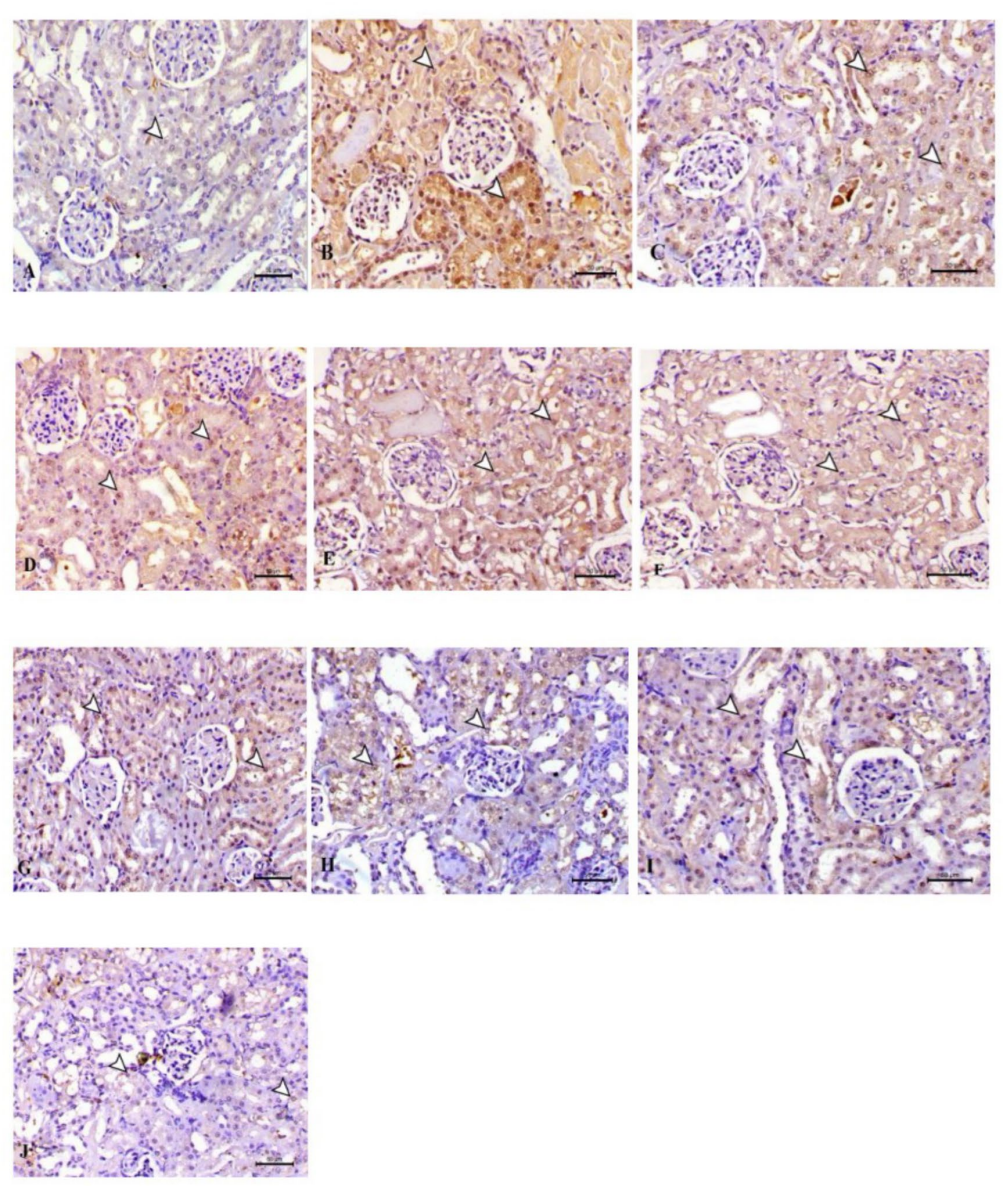
Analysis of caspase 3 in rat kidney using immunohistochemistry. There was no caspase 3 expression in the control group (negative) (**A**). (**B**) The group of rats with gentamicin-induced acute renal failure (ARF) whose renal tubular epithelial cells showed an elevated level of caspase-3 immunoreactivity in their cytoplasm. Caspases-3 are positive when the color is brown. There was no statistically significant increase in caspase-3 immunostaining in the groups treated with nanospanlastic (**D**) and CS (**C**). There was a smaller reduction in caspase-3 immunostaining in the groups treated with GSH and LS (**E**) and (**F**). The immunostaining for caspase-3 was not significantly reduced in the GSH-NPs and LS-NPs groups. Groups I and J, which are combination of GSH-LS and GSH-LS-NPs, exhibited a significant reduction in caspase-3 immunostaining.

**Fig. 14:**
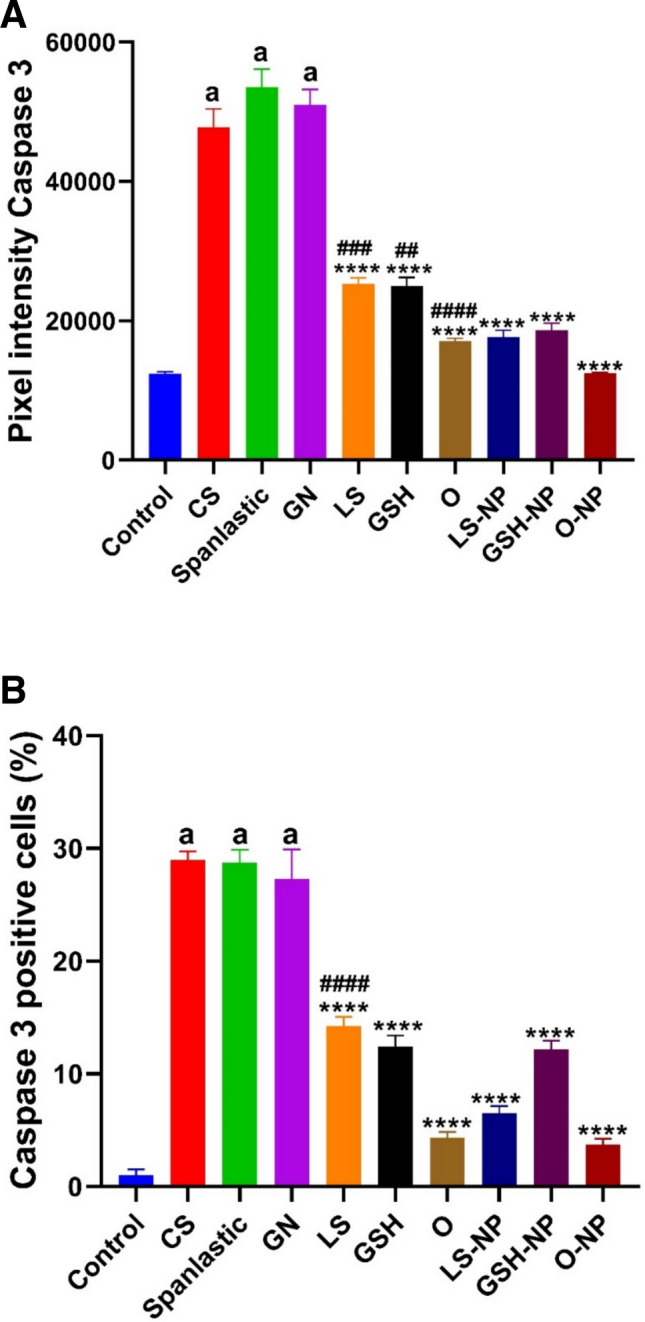
(**A**) Caspases 3 positive cells’ IHC pixel intensity (**B**) The proportion of cells that are immunopositive for caspase 3. *GN* gentamicin, *CS* chitosan, *GSH* glutathione, *LS* (Lepidium sativum), *O* LS-GSH combination, *GSH-NP* glutathione nanoparticles, *LS-NP* *Lepidium sativum* nanoparticles, *O-NP* LS-GSH nanoparticle combinations.

When compared to groups treated with GSH-NPs and LS-NPs, the GN treated group showed a significant increase in caspase 3 immunoreactivity. GSH-LS and GSH-NPs and LS-NPs combinations showed rare expressions, while the control negative group showed no expression (Figs. 13, 14).

## Discussion

Kidney disorders are significant global health issues with significant financial implications. Kidney function can rapidly decrease in ARF, often caused by drug-induced glomerular and tubular nephrotoxicity^3^. Animal models of drug-induced ARF are often established using the aminoglycoside antibiotic GN^32^. GN-induced ARF is characterized by rapid deterioration of kidney function, increased SCr and BUN levels, and albuminuria^2^. Our study found that rats treated with GN showed impaired kidney function, increased SCr and BUN levels, and albuminuria, indicating the development of GN-induced acute renal failure.

The ethanol injection method was employed to prepare LS nanospanlastics using Span 60 and different five edge activators. Span 60 was selected due to its higher transition temperature and biocompatibility when compared with other types^35^. It was documented that spans with high transition temperature provide nanovesicles with high drug encapsulation. In recent years, span 60 was utilized for oral^30^ and transdermal applications^55^. The prepared formulations were found to be in the nanosized range, a crucial parameter for enhancing the efficacy of medicinal products, with a uniform size distribution^38^. The used edge activators have different HLB (hydrophilic lipophilic balance) values that might affect the physicochemical properties of the developed nanospanlastics. The displayed edge activators were Tween 80 (HLB: 15), SDC (HLB: 16.7), Myrj^®^52 (HLB: 16.9), Brij^®^35 (HLB: 16.9), and Cremophor^®^RH40 (HLB: 16). Formulation F1, containing Tween 80, produces smaller vesicles due to increased hydrophobicity and decreased surface energy, resulting in smaller particles compared to other formulas^55^. Zetapotential values above 30 indicate vesicular dispersion colloidal stability, while values over 30 indicate nanosystem dispersions’ stability.

The observed results of measured encapsulation efficiency revealed that all the developed nanospanlastcis exhibited high values of EE%. Whereas the formulation F1 that contains Tween 80 as an edge activator showed a significantly (p < 0.05) higher value of EE% compared to other used edge activators. This might be attributed to the impact of the HLB values of examined edge activators. This result is consistent with previously published reports, which revealed that the lower value of HLB means reduced hydrophilicity and increased the amount of drug that encapsulated into the vesicles, resulting in the higher the encapsulation resulted EE%^35^. Based on formulation F1, which exhibited smaller particle size, higher EE%, and acceptable stability and size distribution, it was selected for further investigations and for In-vivo studies.

The TEM study revealed spherical, nonaggregate vesicle size measurements, smaller than dynamic light scattering results. Zetasizer determined hydrodynamic diameter around vesicles due to water molecules, enlarging size values^56^. The observed results of FTIR revealed the absence of the characteristic peaks of LS in the spectrum of nanospanlastic formulation (F1), confirming the encapsulation of it into nanovesicles^57^. Further, the results of X-ray diffraction suggest that LS is successfully encapsulated into the nanovesicle formulation of Span 60 due to the production of an amorphous structure^55^.

Predicting how well the tested drug nanovehicle will work in vivo required a drug release study, here, the release profile of LS from nanospanlastcis (F1) showed initial rapid release followed by sustained manner for 24 h. This result may be explained by the presence of an unentrapped drug in the outer layer of prepared vehicles. The slower release of LS from F1 compared to free LS solution would be credited to the high transition temperate of Span 60 that offers a more rigid and less permeable vesicle structure, leading to a delayed rate of drug release^57^. The kinetic analysis of release data revealed that the Higuchi diffusion model was the best-suited mechanism of drug release from nanovesicles. The Korsmeyer Peppas equation measured the value of (n) release exponent to understand the mechanism of LS release. The calculated exponent n was found to be 0.55, corresponding to anomalous nonFickian diffusion, whereas the drug release is controlled by diffusion and erosion mechanisms. Consistent with earlier research, these results demonstrated that bilayer vesicles often exhibit prolonged release behavior^30,55^.

According to stability studies for the fabricated nanosystems, it was concluded that nanovesicular carriers may provide a protective core for active products, preventing them from being exposed to environmental conditions. This is in agreement with previous reports^37^ who reported that one potential strategy to improve the physical and chemical stability of active items is to encapsulate them in nanovesicles.

Our results also show that administration of the drugs under research significantly improves albuminuria and glycosuria while also lowering SCr. and BUN levels in rats with GN-induced ARF. These results highlight the validity of these markers as predictors of ARF treatment progress. Results showed a dramatic decrease in kidney damage markers when LS nanoparticulate formulations, GSH monotherapy, or both were compared to their standard drug-treated counterparts. These findings align with those of prior studies^58,59^, who found that LS reduced the detrimental structural and functional effects in rats exposed to cisplatin-induced nephrotoxicity, aluminum chloride -induced nephrotoxicity and dexamethasone-induced nephrotoxicity, respectively. Similarly, in a rat model of diabetic nephropathy, LS reduced urinary albumin excretion and SCr levels^60^. Moreover, GSH treatment was shown to successfully lower SCr and BUN levels in rats exposed to cis-dichlorodiammine platinum (II) in separate investigations conducted by^61^.

Numerous clinical and experimental investigations indicate a strong correlation between GN-induced ARF and oxidative stress^62,63^. Since these findings proposed an interrelationship between oxidative stress and GN-induced ARF, we investigated this hypothesis in our study revealing that oxidative stress was linked to the induction of ARF in rats using GN, as evidenced by elevated renal MDA levels and decreased levels of nitrite and SOD. Interestingly, our therapeutic regimens including LS and GSH as monotherapy and, when used together, in conventional and NP formulations, effectively reduced these harmful signs. Our compounds’ potential therapeutic value in controlling ARF was encouraged by the improved efficacy of the nanoparticulate formulations. Previous research by Abdel-Baky^64^ supports our findings, stating that LS serves as a natural substance for ameliorating the alterations in serum electrolytes, kidney function and oxidative damage in the kidney tissue of rats treated with sodium nitrite. Furthermore, LS has been proven to have antioxidant properties in a rat model of diabetic nephropathy produced by streptozotocin, as demonstrated by a decrease in renal MDA levels and an increase in renal SOD, catalase, and GSH levels^60^. Similarly, GSH was able to reduce cisplatin-induced nephrotoxicity in rats as reflected by decreased levels of MDA and increased levels of GSH^61^.

A safety composite biomarker panel consisting of six new kidney-specific biomarkers was approved by the FDA in 2018 to be used in conjunction with conventional measures to help detect early antibiotic-induced tubular injury because SCr and BUN are neither sensitive nor specific (Chang et al., 2024). NGAL, KIM-1, and serum cystatin C are among these indicators^65^. The most promising indicator of antibiotic-induced nephrotoxicity is serum cystatin C^66,67^. According to Tanase et al.^68^, acute tubulointerstitial nephrotoxicity brought on by xenobiotics, such as cisplatin-induced nephrotoxicity due to oxidative stress, is associated with an increase in urinary KIM-1. As a marker of ARF and a reliable indirect predictor of oxidative stress, NGAL can be detected in serum and urine^69^. Udupa and Prakash^70^ assert that biomarkers generated during tubular injury, such as KIM-1 and NGAL, can be more precise, early, real-time, and proportionate to injury than traditional markers. Alongside with^71,72^, our findings demonstrated that GN treatment markedly raised urine KIM-1 and NAGL and serum cystatin C levels. Additionally, the current study showed that in rats with GN-induced ARF, our treatment regimens, which include solo LS, GSH, and their combination in both NP formulations, significantly reduced serum cystatin C levels and renal tissue concentrations of NGAL and KIM-1, indicating a linked improvement in ARF. Supporting our results, GSH-depleted mice exhibited elevated urinary KIM-1 levels according to a study carried out by Matsubara et al.^73^. To date, no study has used a drug-induced acute renal failure paradigm to investigate the effects of LS on KIM-1 and NGAL levels.

Gentamicin-induced nephrotoxicity triggers inflammation by activating the NF-κβ transcription factor, which subsequently induces the production of pro-inflammatory cytokines IL-6 and TNF-α^74,75^ which in turn can exaggerate oxidative stress by producing more reactive oxygen species^32^. Furthermore, by causing apoptosis and fibrosis, reactive oxygen species and inflammatory cytokines might harm renal tissue^32^. This leads to a significant rise in renal caspase-3 expression, a key facilitator of programmed apoptosis^76^. In alignment with^77,78^, The elevation of the protein expression of IL-6, TNF-α, and caspase-3 in the renal tissue of rats administered GN led to renal inflammation and apoptosis, as demonstrated in our work. It was found that LS had anti-inflammatory properties^15,56^. Also, LS ameliorated the raised levels of pro-inflammatory markers (NF-kβ, IL-1, IL-6, and TNF-α), and the expression level of NF-kβ improving the renal and testicular function in rats with diabetic nephropathy induced by streptozotocin^60,79^. As observed by Junita et al.^80^, in a male rat model of peritonitis, TNF-α levels decreased after GSH injection. Given these facts, our findings may support the idea that giving LS and GSH, in their traditional and NP forms, either alone or in combination, can improve GN-induced ARF by reducing the protein expression of IL-6, TNF-α, and caspase-3. The improvement was more noticeable with the NP versions of GSH and LS.

Interestingly, when compared to the GN-treated group, the carrier NPs-CS and -spanlastics did not significantly modify renal parameters, indicating that they did not worsen damage processes or impair baseline renal function. Thus, their potential safety for drug delivery systems is highlighted by these findings^81,82^. However, a study carried out by Abdel-Wahhab et al.^83^ found that NPs-CS significantly increased glutathione peroxidase and superoxide dismutase levels while decreasing oxidative stress marker levels, MDA, in rats that received ochratoxin A-contaminated diet^84^. Moreover, CS NPs afforded significant protection and amelioration against CCl_4_-induced nephrotoxicity. Thus, CS NPs could afford a potential nanotherapeutic system for the management of nephrotoxicity which allows for broadening their role in biomedical delivery applications^85^.

Acute kidney injury previous investigations have demonstrated that administering gentamicin to rats’ results in glomerular expansion, uneven thickening of the glomerular basement membrane, and neutrophil infiltration, as proven by microscopy^86^. The proximal tubules demonstrate vacuolar degeneration, interstitial edema, and protein deposition^87^. These discoveries align with the findings of our investigation about the histological alterations observed in gentamicin-induced acute renal failure. Moreover, our research indicates that administering LS, GSH, or a combination of both in conventional and NP forms might enhance ARF. Our study’s superior histology results provide evidence for this.

In the progression of kidney disease and fibrosis, caspase-3 plays a crucial role in inducing cell death in renal cells. Hence, we checked caspase-3 levels, a protein involved in cell death. One characteristic of renal cell death and a potential contributor to gentamicin-induced ARF is caspase-3, an essential component of programmed cell death (apoptosis)^88^.

Our study’s immunohistochemical investigation, which agrees with the findings of Abd-Elhamid et al.^89^ and Liu et al.^90^ evidences that caspase-3 protein expression is dramatically upregulated in renal tubular cells during gentamicin-induced ARF. This could provide light on the potential of LS, GSH, or a combination of the two in normal and NP forms to alleviate GN-induced ARF through caspase-3 protein reduction in therapeutic interventions. The benefits of the NP LS and GSH formulations were more noticeable.

In conclusion, biochemical, histopathological, and immunohistochemical results of our study led us to emphasize the reno-protective benefits of LS and GSH, either separately or in combination, when used either traditionally or in conjunction with NPs, can protect kidney structure and function from damage caused by GN. In comparison to the individual drug regimens, concurrent treatment with LS and GSH in their traditional formulations exhibited more significant renal improvement. When used separately or in combination, the NPs versions of these medications showed more beneficial effects than their traditional counterparts. Also, additional research into the possible use of medications loaded with NPs-CS and NPs-spanlastics for the treatment of kidney ailments can be initiated by this work.

## Data Availability

Data and their analysis are provided within the manuscript and related files.

## Abbreviations

ARF: Acute renal failure
BUN: Blood urea nitrogen
CS: Chitosan
EE %: Encapsulation efficiency percentage
FTIR: Fourier Transform Infrared Spectroscopy
GN: Gentamicin
GSH: Glutathione
IL-6: Interleukin-6
KIM-1: Kidney Injury Molecule-1
LS: Lepidium sativum
MDA: Malondialdehyde
NP: Nanoparticle
NGAL: Neutrophil gelatinase-associated lipocalin
PDI: Polydispersity index
SCr: Serum creatinine
SOD: Superoxide dismutase
TEM: Transmission electron microscope
TNF-α: Tumor necrosis factor alpha
